# *APP*-Induced Patterned Neurodegeneration Is Exacerbated by *APOE4* in *Caenorhabditis elegans*

**DOI:** 10.1534/g3.120.401486

**Published:** 2020-06-24

**Authors:** Wisath Sae-Lee, Luisa L. Scott, Lotti Brose, Aliyah J. Encarnacion, Ted Shi, Pragati Kore, Lashaun O. Oyibo, Congxi Ye, Susan K. Rozmiarek, Jonathan T. Pierce

**Affiliations:** Center for Learning and Memory; Waggoner Center for Alcohol and Addiction Research; Cell and Molecular Biology; Department of Neuroscience, The University of Texas at Austin, TX, 78712

**Keywords:** APP, APOE4, *C. elegans*, Neuro-degeneration

## Abstract

Genetic and epidemiological studies have found that variations in the amyloid precursor protein (*APP*) and the apoliopoprotein E (*APOE*) genes represent major modifiers of the progressive neurodegeneration in Alzheimer’s disease (AD). An extra copy of or gain-of-function mutations in *APP* correlate with early onset AD. Compared to the other variants (*APOE2* and *APOE3*), the ε4 allele of *APOE* (*APOE4*) hastens and exacerbates early and late onset forms of AD. Convenient *in vivo* models to study how APP and APOE4 interact at the cellular and molecular level to influence neurodegeneration are lacking. Here, we show that the nematode *C. elegans* can model important aspects of AD including age-related, patterned neurodegeneration that is exacerbated by *APOE4*. Specifically, we found that *APOE4*, but not *APOE3*, acts with *APP* to hasten and expand the pattern of cholinergic neurodegeneration caused by *APP*. Molecular mechanisms underlying how APP and APOE4 synergize to kill some neurons while leaving others unaffected may be uncovered using this convenient worm model of neurodegeneration.

Alzheimer’s disease (AD) is a progressive neurodegenerative disease which cannot be prevented, cured, or decelerated. About 10–20% of people older than 45 in the US are at risk of developing AD in their life (Chêne *et al.* 2015). Genetic and epidemiological studies have associated variation in several genes strongly to AD. Gain-of-function mutations in amyloid precursor protein (*APP*) or in presenilin genes related with processing APP are associated to early-onset AD (Goate *et al.* 1991). Possession of extra wild-type copies of *APP*, such as found in Down Syndrome (DS) or in rare individuals without DS, is also associated to early-onset AD (Prasher *et al.* 1998; Cabrejo *et al.* 2006). Additionally, a distinct variant of *APP* appears protective against AD (Jonsson *et al.* 2012). The strongest genetic risk factor for the more common late-onset AD is the ε4 allele of apolipoprotein E (*APOE4*) (Corder *et al.* 1993; Liu *et al.* 2013). Over 65–80% of AD patients carry an *APOE4* allele (Saunders *et al.* 1993; Farrer *et al.* 1997). The lifetime risk for AD in people homozygous for *APOE4* is extremely high (91%) relative to those with the more common *APOE3* allele (20%) or the less common *APOE2* allele (2.8–4.5%) (Corder *et al.* 1993; Corder *et al.* 1994). *APOE4* is associated with more pronounced neurodegeneration (Holtzman *et al.* 2012); consequently, each copy of *APOE4* predicts shorter lifespan relative to the more common *APOE3* variant (Liu *et al.* 2013). Even for early-onset AD, the *APOE4* variant is associated with earlier onset and harsher severity, including elevating levels of the amyloid-β_1-40_ peptide and faster spread of neurodegeneration in the case of Down syndrome (Patel *et al.* 2011; Head *et al.* 2011). This suggests that *APP* and *APOE4* may contribute an additive or synergistic risk for AD onset and progression.

Despite the substantial influence of *APOE4* on the progression of AD, how APOE4 modulates the molecular mechanisms underlying neurodegeneration remains elusive. Study of genetically modified mice has contributed to our understanding of the susceptibility to AD and other neurodegenerative diseases conferred by mutations or variants in *APP*, *APOE*, and related genes (Di Battista *et al.* 2016). However, progress toward understanding the mechanistic underpinnings of some AD-related pathologies, particularly neurodegeneration, has been slower (LaFerla and Green 2012). Many mouse models of AD do not show neurodegeneration that is central to the human condition (Jankowsky and Zheng 2017). Moreover, it is more expensive and time-consuming to study age-related diseases in mice.

To surmount some of these limitations, we explored whether *APOE* could be studied in the context of *APP*-related neurodegeneration with the genetically tractable model nematode, *Caenorhabditis elegans* (Yi *et al.* 2017). This minimal *in vivo* animal model offers several advantages for basic and applied research for AD. First, worms mature rapidly to adulthood, reaching “middle-age” of adulthood in only 5 days as reproduction declines. Despite this compressed lifespan, *C. elegans* shares many of the genetic, cellular, and molecular processes of aging with humans and mice (Arey and Murphy 2017). Thus, age-dependent processes can be studied within the span of one week with *C. elegans*. Second, forward and reverse genetics as well as transgenesis studies are extremely rapid with *C. elegans*. Third, every cell in the tiny (1 mm) worm is identified, including its 302 neurons, which can be examined individually using fluorescent reporters in the living, transparent worm. The function and health of even single identified neurons can be further probed by quantifying simple behaviors such as egg laying and locomotion. Fourth, several models of neurodegeneration related to AD have been generated using *C. elegans* (Griffin *et al.* 2017). We recently described a transgenic worm strain that expresses a single copy of human *APP* and displays degeneration of a subset of cholinergic neurons in middle-age adulthood (Yi *et al.* 2017; Mondal *et al.* 2018). Using this *APP*-expressing strain, we discovered small molecules that both prevent degeneration of neurons in worm via a conserved signaling pathway and boost cognition in a mouse model of AD (Yi *et al.* 2017). Thus, although the simple worm cannot be used to study the important decline in cognition and memory as in mouse models, it can be used to directly study degeneration of identified neurons and to indirectly predict effective pharmacological approaches in rodent models of AD.

In this study, we tested how expressing variants of human *APOE* altered health and function of neurons in our worm model of *APP*-related neurodegeneration. We observed several key characteristics associated with AD. First, *APOE4* caused a higher level of neurodegeneration than *APOE3* and, *APOE4* but not *APOE3* acted in concert with *APP* to further increase neurodegeneration. This retains the variant-specific effect of *APOE* that is well-documented in humans (Corder *et al.* 1993). Second, *APOE4* accelerated neurodegeneration in *C. elegans*, lowering the age of onset from late to early adulthood. This mirrors the expedited onset of degeneration in humans who carry *APOE4* (Holtzman *et al.* 2012). Lastly, despite deliberate expression of *APP* and *APOE4* throughout the nervous system, the pattern of neurodegeneration in the worm model was restricted. This result is similar to the initially restricted degeneration of specific brain regions, *i.e.*, the hippocampus and the entorhinal cortex, despite the near-ubiquitous expression of APP and APOE in the brain seen in AD patients (Holtzman *et al.* 2012). Because *APOE* variant expression influences *C. elegans* neurodegeneration in a manner similar to several key aspects found in human AD, our worm model may facilitate both the discovery of molecular pathways involved in the development of AD as well as novel drugs for the treatment of AD.

## Materials and Methods

### Plasmid and transgenic animals

The plasmid constructs for human *APP* or *APOE* transgenes under a pan-neuronal promoter (*prab-3*::*APP*::*mCherry*::*UNC-54* UTR; *prab-3*::*APOE4*::*UNC54* UTR; *prab-3*::*APOE3*::*UNC54* UTR) were generated using Multi-site Gateway technology (Invitrogen, Carlsbad, CA). Middle entry clones were made using the MultiSite Gateway Three-Fragment Vector Construction Kit (Invitrogen). cDNA for human *APP695* or *APOE* variants (*APOE3* or *APOE4*) was amplified using the High Fidelity Phusion Polymerase (NEB) and recombined into pDONR221 using the BP clonase (Invitrogen). To generate the expression clones, the middle entry clones were previously fused into pCFJ150 along with the 5′ *rab-3* pan-neuronal promoter and 3′ *unc-54* UTR entry clones (Yi *et al.* 2017) using the LR clonase II (Invitrogen). Resulting plasmids were verified by sequencing. The construct for the GFP reporter for HSN neurons was made by a PCR fusion of a ∼2 kb region upstream of tph-1 amplified from genomic DNA and *GFP*::*unc-54* UTR amplified from pPD95.75. The construct for the GFP reporter for VA and VB neurons was made by a PCR fusion of a ∼1.8 kb region upstream of *del-1* amplified from genomic DNA and *GFP*::*unc-54 UTR* amplified from pPD95.75. The constructs to express *APOE4* in the intestine and coelomocytes were made via NEBuilder HiFi DNA Assembly. The promoter of *fat-7* (∼1.2 kb upstream of the gene) was amplified from genomic DNA while the promoter of *unc-122* (∼300 bp) was amplified from pCFJ68.

Single copy transgenic strains for human *APP* and *C. elegans **apl-1* were generated via MosSCI methods as described by Frøkjaer-Jensen *et al.* (2008). A single copy of human *APP* or *apl*-1 under a *rab-3* promoter was inserted into the chromosome II. The EG4322 strain was selected for the insertion of the construct at ttTi5605 II. Insertion of *APP* or *apl-1* was confirmed by PCR and sequencing of the modified region on the chromosome. Extrachromosomal array strains in this study were made by injecting expression plasmids and co-injection markers (1.2 ng/μl for pCFJ90, *pmyo-2:mCherry*, and 30 ng/μl for pCFJ68, *punc-122*::*GFP*, *ptph-1*::*GFP*) described by (Mello *et al.* 1991). The extrachromosomal array for *ptph-1*::*GFP* was subsequently integrated using standard UV-integration techniques and outcrossed 6 times; this JPS617 strain served as the wild-type (WT) background for all other strains.

We made three attempts to integrate the extrachromosomal array expressing *APOE4* using traditional UV-integration techniques. However, the attempts were unsuccessful, at least in part due to a low survival rate after UV exposure. Instead, the array [*prab-3*::*APOE4*::*UNC54 UTR*; *pmyo-2:mCherry*] was integrated using CRISPR-cas9-based methods modified from Yoshina *et al.* (2015). Briefly, two plasmids containing the *cas9* gene (pDD162) and a guide RNA targeting either the genomic region of integration (LG X:22.84, a location previously used for MosSCI insertions (Frøkjaer-Jensen *et al.* 2008)) or a region on the extrachromosomal array (β-lactamase gene), and a co-injection marker (pCFJ104, *pmyo-3:mCherry*) were injected into worms carrying the *APOE4* array. The sequences for guide RNAs used in the integration are as follow, 5′TTAATAGACTGGATGGAGG3′ (β-lactamase gene), 5′ATGTGTCATAAGTCAACAAC3′ and 5′TTATGTAGTCTCTTTCAGTG3′ (X:22.84). The guide RNAs were made according to Ward (2015). After integration of the linearized array, we chose a strain with a similar intensity of red pharynx as that seen prior to integration. The same method was employed by SunyBiotech (Fuzhou City, China) to generate an integrated APOE3 strain PHX2443 *sybIs2443[prab-3*::*ApoE3*::*unc-54 UTR*, *pmyo-2*::*mCherry*::*unc-54 UTR]*.

The integrated *APOE4* and *APOE3* lines were crossed to JPS617 to make the APOE4 (JPS844) and APOE3 (JPS1312) strains with GFP-labeled VC and HSN neurons. JPS844 was then crossed to JPS809 to make APOE4+APP (JPS845) strains with GFP-labeled VC and HSN neurons. Subsequently, JPS845 was crossed with MT3002 (*ced-3**(**n1286**)* IV) to obtain an APOE4+APP strain with a *ced-3* null background. The genotypes for all the strains used in this study are listed in Table S1.

### Bag of worms assay

Worms were maintained at 20° as previously described by Brenner (1974). A total of 50 worms were age-synchronized by picking L4-stage larvae onto NGM agar plates seeded with OP50 bacteria. Adult worms were carefully examined every day for up to four days (Day 1-4 of adulthood) for the bag-of-worms (BW) phenotype indicated by the presence of hatched larvae within their body. Groups of healthy worms with no hatched larvae were transferred to a freshly seeded plate each day. The reported percentage BW represents the number of BW observed over the period of observation (2-4 days) relative to the total number of assayed worms. The assay was repeated 3-4 times for each genotype at different times to control for conditions that may vary by date. BW phenotype was quantified as a percentage ± 95% confidence interval. Group comparisons were made with planned *χ^2^*-tests.

### Scoring of neurodegeneration

**Figure 1:**
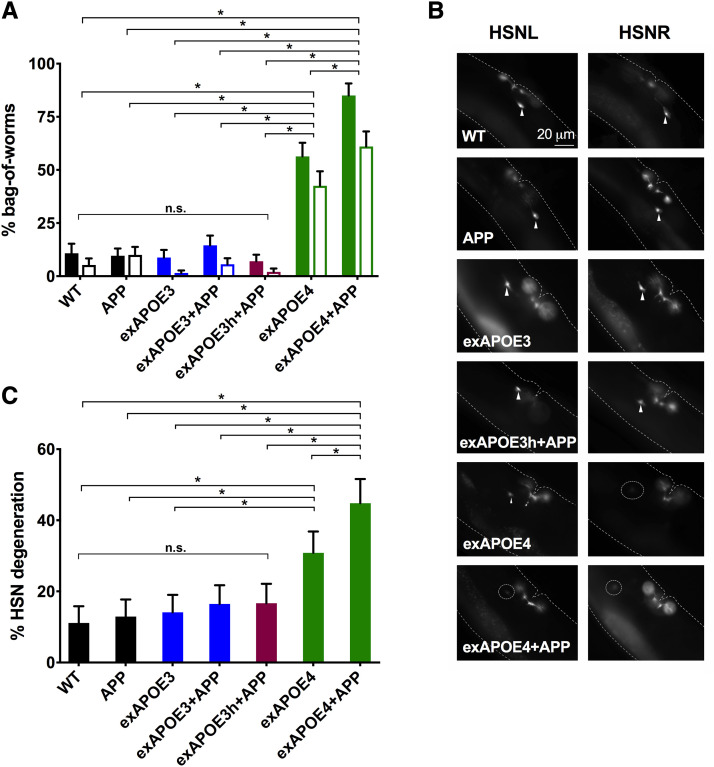
Pan-neuronal expression of *APOE4* induces neurodegeneration in HSN neurons that is exacerbated by *APP*. *A*, Histogram shows the cumulative percent bag-of-worms (BW) phenotype during the first 3 days of adulthood. BW was determined by the presence of progeny with pharyngeal mCherry expression in a hermaphrodite. Two strains of each genotype (signified by grouped pairs of solid and open bars) were made with independent extrachromosomal arrays containing a *pmyo-2*::*mCherry* reporter. The *APP* transgene was integrated in all *APP* strains. The BW phenotype was frequently observed in *APOE4* strains with or without *APP* but not in strains with *APP* alone. In contrast, *APOE3* transformed at the same concentration as *APOE4* (exAPOE3) or double the concentration (exAPOE3h), did not increase the frequency of BW. For statistical comparisons, solid and open bars were each treated as a set. Within a set, all pairwise comparisons were made with χ^2^ tests. Alpha was set at 0.001 to correct for multiple comparisons (*P < 0.001). *B*, Fluorescent images of the neurons, HSNL and HSNR, in Day 3 adults. Many *APOE4* worms with or without *APP* showed morphological abnormalities or a total loss of one or both HSN neurons. Arrowheads indicate healthy HSN neurons. Dotted circles indicate degenerated neurons. *C*, Histogram shows the percent HSN neurodegeneration on Day 3 of adulthood for a set of strains assayed in *A*. Pairwise comparisons were made with χ^2^ tests. Alpha was set at 0.003 to correct for multiple comparisons (*P < 0.003).

**Figure 2:**
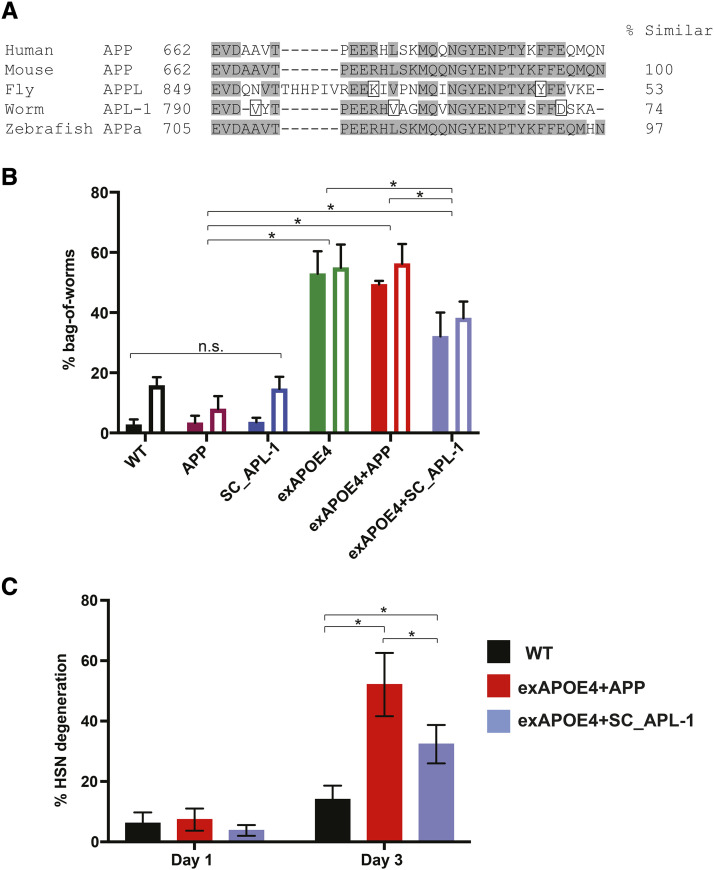
Overexpression of APP-related *C. elegans* gene *apl-1* does not mimic APP in elevating APOE4-induced neurodegeneration. *A*, Species alignment to the 31 residues of the human APP intracellular domain (AICD). Shaded residues are identical to human sequence, and boxes indicate conservative changes. *B*, Single-copy (SC) overexpressed *apl-1* is protective against bag-of-worms phenotype in an *APOE4* background. Histogram showing the cumulative percent bag-of-worms phenotype during the first 4 days of adulthood. Bag-of-worms was determined by visualization of larval worm within the body of a hermaphrodite under stereomicroscope. Solid and open bars represent trials compared to different SC *apl-1* controls. For statistical comparisons, solid and open bars were compared to their respective controls. Comparisons were made with χ^2^ tests. *C*, APOE4-induced HSN neurodegeneration is diminished in SC *apl-1* background. Histogram showing the percent HSN neurodegeneration for Day 1 and Day 3 adults. Comparisons were made with χ^2^ tests.

**Figure 3:**
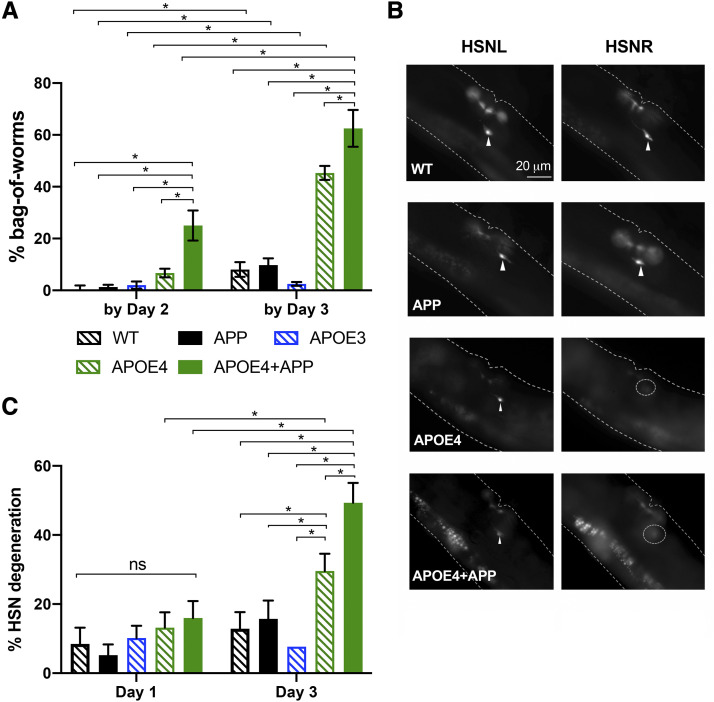
Pan-neuronal co-expression of *APOE4* and *APP* induces age-related neurodegeneration in HSN neurons, not seen with *APOE3*. *A*, Histogram shows the cumulative percent bag-of-worms (BW) phenotype by Day 2 and Day 3 of adulthood. The frequency of the BW increased at an earlier age for the APOE4+APP strain than for the other genotypes. *APP*, *APOE3*, and *APOE4* transgenes were integrated. *B*, Fluorescent images of the neurons, HSNL and HSNR, in Day 3 adults. Integrated strains were scored based on a total loss of one or both HSN neurons. Arrowheads indicate healthy HSN neurons. Dotted circles indicate degenerated neurons. Dimmer GFP expression in the integrated *APOE4* strains (JPS844 and JPS845) made it difficult to see the HSN processes; therefore neurodegeneration was scored as the absence of HSN cell bodies in these strains. *C*, Histogram shows the percent HSN neurodegeneration for Days 1 and 3 adults. For *A* and *C*, pairwise comparisons within day and within-strain across days were made with χ^2^ tests. Alpha was set at 0.003 to correct for multiple comparisons (*P < 0.003).

Worms were maintained at 20° and synchronized by picking L4-stage larvae onto NGM plates seeded with OP50 bacteria and containing 0.35-mM 5-fluoro-2’-deoxyuridine-5′-phosphate (FUDR) to sterilize adults and prevent non-specific consequences of larvae hatching inside adults. Prior to scoring neuron health, worms were cleaned by transferring them to an unseeded plate until they left no residual tracks of bacteria, a process that took ∼10 min. Worms were mounted on 2% agarose pads, immobilized with 30-mM sodium azide and imaged on an Olympus IX51 inverted microscope equipped with an X-Cite FIRE LED Illuminator (Excelitas Technologies Corp.) and an Olympus UPlanFL N 40X/0.75 NA objective. Epifluorescence images were taken with a Retiga 2000R CCD camera (QImaging) and QCapture Pro 6.0 software. For Figures 2C and 3C worms were identically maintained, but immobilized mechanically without azide on a microfluidic vivoChip (Newormics, Austin, TX) in filtered M9 buffer and imaged at 40X. For strains with no or extrachromosomal expression of *APOE* (Figure 1), HSN neurons were considered healthy when the scored HSN cell body was clearly present and the processes were intact. HSNs were considered degenerated when the scored neuron was absent and/or displayed significant morphological abnormalities (*e.g.*, blebbing, beading and absence of processes). Examples of degenerated HSN neurons following these criteria can be found in Fig. S1. We observed dimmer GFP expression in the HSNs of integrated *APOE4* strains (Figure 3) making it more difficult to visualize the HSN processes. Thus, HSN health in these strains (JPS844 and JPS845) was based exclusively on the presence or absence of the HSN cell-bodies. That is, HSNs were considered to be degenerated when the scored HSN neurons were absent. Neuron health was scored with the observer blind to genotype. An average of 50 worms were scored per strain for each trial. Scoring was repeated 3-4 times for each genotype and expressed as percent HSN degeneration ± 95% confidence interval. Group comparisons were made with planned *χ^2^*-tests.

### Locomotion assay

Worms were cleaned of bacteria as described above. Approximately 15 worms were moved into a 5/8-inch-diameter copper ring sealed on a standard unseeded NGM agar plate. Movement was recorded for 2 min at 2 frames/sec with a FLEA digital camera (Point Gray, Richmond, BC, Canada). The distance that the worms crawled during 1 min was measured using a semi-automated procedure in ImagePro Plus (Media Cybernetics, Rockville, MD) to objectively calculate overall speed of individual worms. Speed for each group was quantified as group mean ± SEM. Group comparisons were made with planned Student’s *t*-tests.

### Semi-quantitative PCR

Five worms of each genotype (WT, integrated *APOE3*, integrated *APOE4*, extrachromosomal *APOE3*, extrachromosomal *APOE4*) were lysed and digested in buffer + proteinase K for one hour. PCR was performed using 2 μl of template for each reaction with 2x DreamTaq Green Master Mix (FisherSci) and primers: forward 5′-CGGACATGGAGGACGTG-3′, reverse 5′-AGCCGCTTACGCAGCTTG-3′. Samples were run on a 1.5% agarose gel at 150V for 40min and imaged on a BioRad UV Imager. Images were exported into Image Lab (BioRad), where lanes and bands were manually selected, and band intensity was measured. Adjusted band intensities were exported into Prism 8 (GraphPad), and data are presented as mean ± SEM. Three independent biological replicates were measured for each genomic preparation.

### RT-qPCR

Worms were washed from near-starved plates (3 plates for WT and integrated, 9 plates for extrachromosomal) and pelleted. Each pellet was flash frozen in liquid nitrogen, thawed on ice, and vortexed 6 times. The pellet was further disrupted by needle and syringe in RNA extraction buffer. RNA was isolated (Zymo *Quick*-RNA Miniprep; QIAGEN RNeasy Mini Kit), and 1ug was reverse transcribed into cDNA with the Protoscript II cDNA synthesis kit (NEB). qPCR was performed using SYBR Green (FisherSci) on a Roche 480 Lightcycler (Roche) using primers: *APOE* forward 5′-CCTGGACGAGGTGAAGGAGCA-3′, reverse 5′-CTCGAACCAGCTCTTGAGGC-3′ and *tba-1* forward 5′-ATCTCTGCTGACAAGGCTTAC-3′, reverse 5′-GTACAAGAGGCAAACAGCCAT-3′. Ct values were exported and analyzed using the ΔCt method. No values were detected for no template and no RT controls. Data represents the mean of two biological replicates with three technical replicates each. The housekeeping gene *tba-1* was used as a control, and relative expression was normalized to this reference gene.

### Data availability

Strains and plasmids are available upon request. The authors affirm that all data necessary for confirming the conclusions of the article are present within the article, figures, and tables. Table S1 contains genotypes for each strain used in this study. Supplemental material available at figshare: https://doi.org/10.25387/g3.12493769.

## Results

### APP expression exacerbates APOE4-induced neurodegeneration in HSN neurons

We previously developed a *C. elegans* model of neurodegeneration that expressed a single-copy of human *APP* and showed age-related degeneration of a subset of cholinergic neurons (Yi *et al.* 2017; Mondal *et al.* 2018). Specifically, six VC-class neurons located in the middle of the ventral nerve cord begin to die as the worm advances past Day 3 of adulthood. To investigate whether variants in *APOE* modify neurodegeneration phenotypes in our APP worm model, we generated strains that expressed human *APOE4* or *APOE3*, with or without human *APP*. Both *APP* and *APOE* transgenes were expressed throughout the nervous system using the conventional pan-neuronal *rab-3* promoter to mimic widespread brain expression in mammals (Stefanakis *et al.* 2015). *APP* was tagged with mCherry at the C-terminus and was found to be expressed throughout the nervous system of the worm (Fig. S2). *APOE* expression was verified via RT-qPCR (Fig. S3). *APP* was integrated while initially *APOE* transgenes were expressed with extrachromosomal arrays. Comparisons were made between these strains and a wild-type background (WT). While studies have shown a neuroprotective effect of APOE2 in humans and other models (Conejero-Goldberg *et al.* 2014; Wu and Zhao 2016; Griffin *et al.* 2019; Reiman *et al.* 2020), our study focused only on the effects of human *APOE3* or *APOE4* expression.

Despite the degeneration of VC neurons in our *APP*-expressing strain, the expression of human *APP* alone did not confer any obvious behavioral defects (Mondal *et al.* 2018). To test if *APOE* variants caused gross phenotypic differences that may be indicative of neuronal degeneration or dysfunction beyond that observed in our strain only expressing *APP*, we evaluated the behavior of *APOE3*- or *APOE4*-expressing strains. We observed an increase in a behavioral phenotype called ‘bag-of-worms’ (BW) in strains expressing *APOE4* but not *APOE3* (Figure 1A). *C. elegans* normally lays eggs that hatch outside the parent. However, eggs are retained inside the parent under stressful conditions of starvation, or when the neurons that mediate egg-laying become dysfunctional or die (Schafer 2005). If eggs are retained too long in the worm, they hatch and fill the parent with writhing larvae (Angelo and Van Glist 2009). This BW phenotype can be unambiguously detected at low power via a stereomicroscope and has proven convenient to study many biological processes (*e.g.*, Trent *et al.* 1983; Conradt and Horvitz 1998).

When we quantified the amount of BW by observing worms for 3 days throughout the *C. elegans* reproductive period, we found that WT worms displayed a low level of BW as expected. The incidence of BW was not raised in strains expressing *APP* or *APOE3*. However, *APOE4*-expressing strains had a significantly higher percentage of BW than WT, *APP*-, and *APOE3*-expressing strains (Figure 1A). Worms expressing both *APOE4* and *APP* (APOE4+APP) displayed an even higher incidence of BW than worms expressing either *APOE4* or *APP* alone (Figure 1A). In contrast, the percentage BW in strains expressing APOE3+APP remained low and was not significantly higher than that found in strains expressing either gene individually (Figure 1A). The lack of effect of *APOE3* could not be easily explained by a lower number of *APOE3* transgene copies on the extrachromosome *vs.* *APOE4* copies because an independent strain made with a higher (2x) transformative dose (exAPOE3h) showed a low level of BW indistinguishable from the APP strain (Figure 1A).

Because the *APOE4*-expressing strains displayed the BW phenotype even when well-fed, this suggested that one or more of the egg-laying neurons were dysfunctional or dying. Egg-laying is controlled primarily by the left-right pair of HSN neurons and to a much lesser extent by VC4 and VC5 neurons (Schafer 2005). The HSN and VC4 and VC5 cholinergic neurons connect reciprocally, as well as to egg-laying muscles by synapses (Altun and Hall 2018). We checked the health of these neurons by directly visualizing them with a fluorescent reporter through the worm’s transparent body (see Methods). We considered HSN neurons “degenerated” when the neuron was absent and/or displayed significant morphological abnormalities (*e.g.*, blebbing, beading and absence of processes) (see Figures 1B and S1). Additionally, we saw clear degeneration of HSN neurons in *APOE4*-expressing strains with or without co-expression of *APP*. Consistent with our observed BW behavior, the incidence of HSN neuron degeneration was significantly higher in APOE4+APP worms than those expressing either *APP* or *APOE4* alone (Figure 1C). Intriguingly, although expression of *APOE4* increased degeneration of HSN neurons, the expression of *APOE3* with or without *APP* co-expression did not increase HSN degeneration relative to WT (Figure 1C). Even when the *APOE3* transgene was transformed at double the concentration of the *APOE4* transgene and combined with *APP* expression, the resulting strains exhibited a percentage BW and HSN neurodegeneration that was not significantly higher than WT (Figure 1A,C).

Reminiscent to our previous findings with APP alone, APOE4-induced neurodegeneration increased in an age-dependent manner, from few at Day 1 of adulthood to many by Day 3 of adulthood (Yi *et al.* 2017; Mondal *et al.* 2018). As BW behavior results in death of the adult, our sample size was too diminished to study older (> day 4) adults.

Others have found that APOE4 may worsen outcomes in AD patients and models via several mechanisms, including some that depend on APP. For instance, APOE4 exacerbates Aβ deposition in transgenic models, though a direct interaction between APOE4 and APP has not been shown (Ye *et al.* 2005; Bales *et al.* 2009; Bien-Ly *et al.* 2011; Kim *et al.* 2011; Liu *et al.* 2017). Although *C. elegans* does not have a clear ortholog of *APP*, it has a related gene, *apl-1*, with 71% sequence similarity to the intracellular domain of APP (Figure 2A; Daigle and Li 1993). *C. elegans* lacks a clear β-secretase ortholog, suggesting an Aβ-like fragment would not be processed from either human APP or APL-1. Importantly, APL-1 has no Aβ domain. Nevertheless, we decided to test whether APOE4-induced degeneration of HSN neurons depended on *apl-1*. However, we could not easily test whether APOE4-induced neurodegeneration required *apl-1* by knocking it out, because *apl-1* is essential for development (Hornsten *et al.* 2007). Instead, we tested whether knocking in an extra copy of *apl-1* mimicked the synergistic effects of knocking in *APP*. Specifically, we integrated a single additional copy of *apl-1* expressed pan-neuronally and tagged with mCherry (SC_APL-1). The SC_APL-1 strain also served as a control for knocking a single mCherry-tagged transgene into a specific locus on the second chromosome in our APP model.

We generated strains expressing both *SC_apl-1* and *APOE4* and tested for BW and HSN neurodegeneration. Overexpression of *apl-1* alone did not result in an incidence of BW above WT levels. Interestingly, expression of *SC_apl-1* with *APOE4* showed an unexpected, significant reduction in BW compared to *APOE4* alone (Figure 2B) suggesting that HSN function was retained. Consistent with these behavioral results, we found that HSN neurodegeneration was reduced in APOE4+SC_APL-1 strains compared to APOE4+APP strains (Figure 2C). We conclude that the BW and neurodegeneration phenotypes seen in our *APOE4*-expressing strain is not exacerbated by over-expression of *apl-1*. This suggests that APOE4 likely does not interact with APL-1 in the same way it does with APP to give rise to these phenotypes.

Taken together, these results demonstrate that the pan-neuronal expression of *APOE4* causes degeneration of the HSN neurons which happen to synapse with VC neurons. Further, co-expression of *APOE4* with *APP* extends the level of HSN neurodegeneration beyond that observed when either gene is expressed singly.

### Neuronal APOE4 causes selective neurodegeneration

Thus far, we used extrachromosomal arrays to express the *APOE* transgenes. Although extrachromosomal arrays represent a convenient approach to express transgenes, these arrays are not perfectly carried through cell division which gives rise to mosaic individuals (Leung-Hagesteijn *et al.* 1992). Moreover, the expression of extrachromosomal transgenes is sometimes suppressed (Hsieh and Fire 2000). To control for these potential caveats, we sought to integrate the *APOE3* and *APOE4* transgene arrays into the worm’s X chromosome and re-evaluate BW phenotype and HSN degeneration. In this way, the worm would be expected to faithfully express the *APOE3* and *APOE4* transgenes throughout the nervous system.

We again assayed BW and HSN neurodegeneration, breaking down the incidence as a function of age. The integrated APOE4 and APOE4+APP strains retained a comparable percentage of BW as observed in the extrachromosomal array strains by Day 3 of adulthood, while the integrated APOE3 strain did not exhibit significant phenotypes (Figure 1A *vs.* Figure 3A). Interestingly, not only was the total incidence of BW higher in the APOE4+APP strain by Day 3 of adulthood, but the total incidence of BW was already substantially raised by Day 2 of adulthood relative to that seen in the APOE4 strain (Figure 3A). Though both of the APOE4 and APOE4+APP strains showed age-dependent degeneration of HSN neurons, the degree of degeneration was highest in the APOE4+APP strain (Figure 3C). These results are consistent with our findings for the extrachromosomal strains. Differences in how APOE4 but not APOE3 strains displayed degeneration could not be simply attributed to higher levels of expression of the APOE4 transgene compared to APOE3. Both semi-quantitative genomic PCR and RT-qPCR revealed subtle but no significant differences in expression of both integrated and extrachromosomal transgenes (Fig. S3).

**Figure 4:**
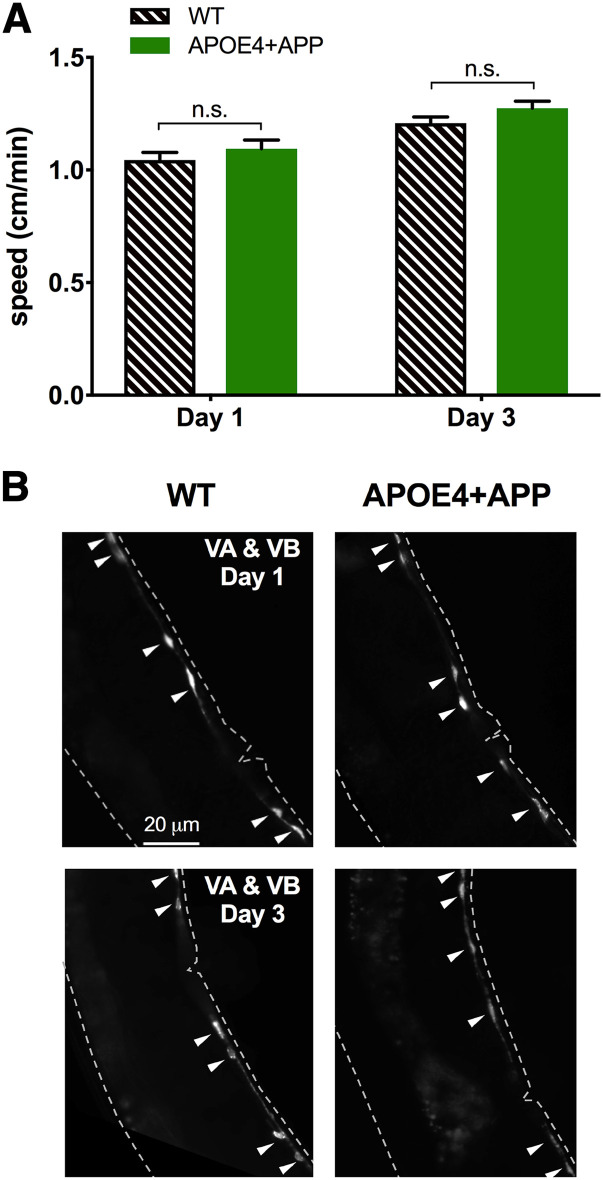
Neurodegeneration induced by pan-neuronal co-expression of *APOE4* and *APP* spares VA and VB neurons. *A*, Locomotion, a behavior mediated in part by VA and VB neurons, is not disrupted in APOE4+APP worms compared to WT worms. Histogram showing mean crawl speed ± SEM for WT and APOE4+APP worms on Days 1 and 3 of adulthood. Strain comparisons were made using Student’s *t*-tests. *B*, Fluorescent images of the VA and VB neurons show no morphological abnormalities in Day 3 APOE4+APP adults relative to Day 1 APOE4+APP or WT adults. Arrowheads indicate healthy VA and VB neurons. Both *APP* and *APOE4* transgenes were integrated.

When we expressed *APOE4* and *APP* throughout the nervous system, we originally expected to observe gross defects across multiple behaviors. However, aside from the BW phenotype, no gross movement defects were apparent in the APOE4+APP strain. Moreover, APOE4+APP worms exhibited no difference in crawling speed compared to WT worms (Figure 4A. The crawling speed was indistinguishable on both Day 1 and Day 3 of adulthood between WT and APOE4+APP worms (Figure 4A). Consistent with this behavioral observation, when we looked at GFP-labeled VA and VB cholinergic motor neurons, which contribute to locomotion, we saw no qualitatively discernable differences in morphology and number of VA and VB neurons between WT and APOE4+APP worms (Figure 4B).

**Figure 5:**
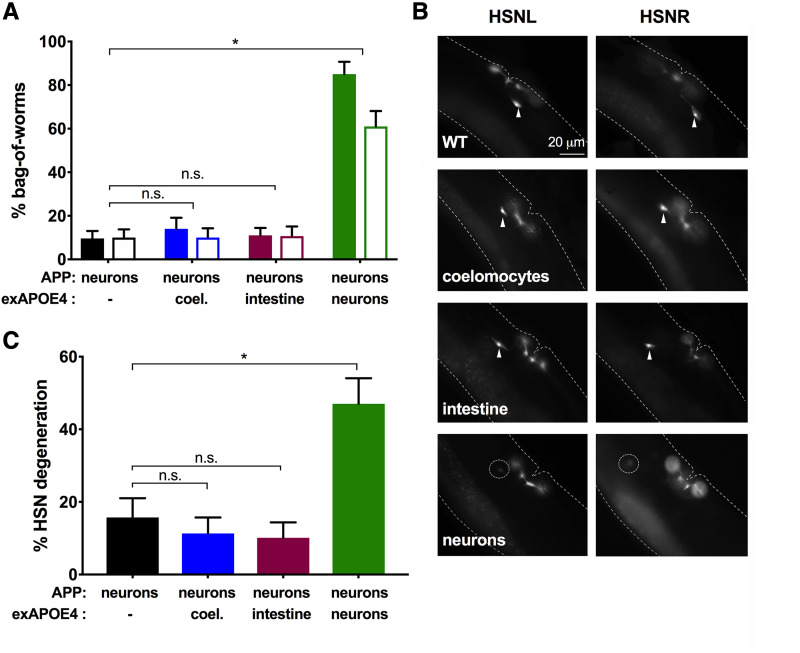
Pan-neuronal, but not non-neuronal, expression of *APOE4* promotes HSN neurodegeneration in a pan-neuronal *APP* background. *A*, Histogram shows the cumulative percent bag-of-worms (BW) phenotype by Day 3 of adulthood. The frequency of the BW increased in worms with pan-neuronal *APP* when *APOE4* was expressed in neurons, but not in coelomocytes or intestine. Two strains of each genotype were made (signified by groups of paired solid and open bars) with independent extrachromosomal arrays containing a *pmyo-2*::*mCherry* and *APOE4* transgene. The *APP* transgene was integrated. For statistical comparisons, shaded bars and open bars were each treated as a set. Within a set, each exAPOE4 strain was compared with the background APP strain using χ^2^ tests. Alpha was set at 0.008 to correct for multiple comparisons (*P < 0.008). *B*, Fluorescent images of the neurons, HSNL and HSNR, in Day 3 adults. Many worms expressing pan-neuronal *APOE4* on the *APP* background show morphological abnormalities or a total loss of one or both HSN neurons. Label indicates the location of *APOE4* expression. Arrowheads indicate healthy HSN neurons. Dotted circles indicate degenerated neurons. *C*, Histogram showing the percent HSN neurodegeneration on Day 3 of adulthood for a set of strains assayed in *A*. Each exAPOE4 strain was compared with the background APP strain using χ^2^ tests. Alpha was set at 0.015 to correct for multiple comparisons (*P < 0.015).

Next, we asked whether *APOE4* induces neurodegeneration when expressed outside the nervous system in a pan-neuronal *APP* background. Using extrachromosomal arrays, we expressed *APOE4* in organs involved in metabolic and excretory functions, the intestine and coelomocytes, using the *fat-7* and *unc-122* promoters, respectively. Unlike with pan-neuronally expressed *APOE4*, the incidence of BW was as low for strains expressing *APOE4* in either coelomocytes or intestine as in the background *APP* strains (Figure 5A). This low percentage of BW for strains with *APOE4* expressed in coelomocytes or intestine corresponded with low levels of HSN degeneration in these strains (Figure 5B,C).

**Figure 6:**
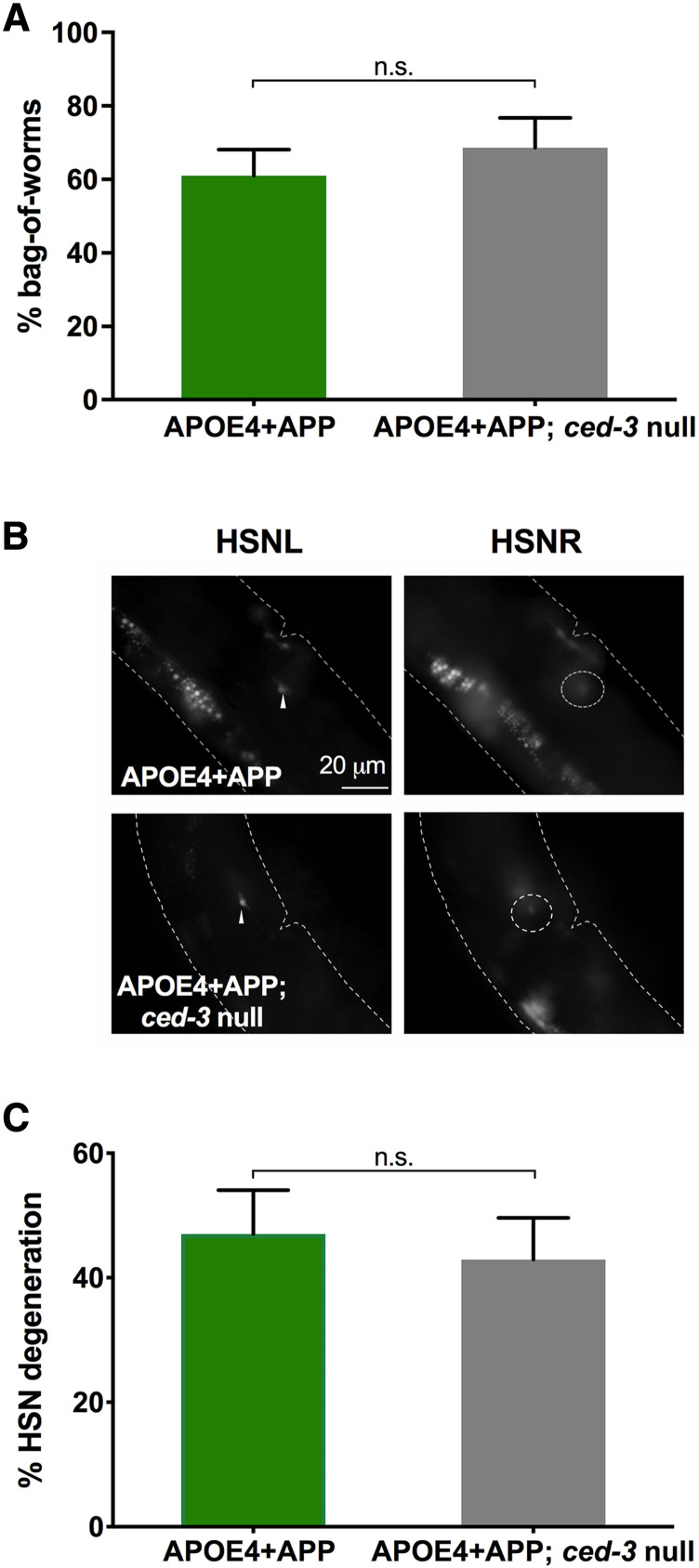
Neurodegeneration induced by pan-neuronal co-expression of *APOE4* and *APP* is not mediated by CED-3. *A*, Histogram shows the cumulative percent bag-of-worms (BW) phenotype by day 3 of adulthood. The frequency of BW in the APOE4+APP strain is not altered in a *ced-3* null background. Both *APP* and *APOE4* transgenes were integrated. *B*, Likewise, *ced-3* expression does not alter neurodegeneration in the APOE4+APP strain. Fluorescent images of HSNL and HSNR. Arrowheads indicate healthy HSN neurons. Dotted circles indicate degenerated neurons. *C*, Histogram showing the percent HSN neurodegeneration for Day 3 adults. Comparisons were made with χ^2^ tests.

To better understand the mechanism of degeneration, we investigated whether HSN neurons degenerate in a manner dependent on apoptosis. We crossed the *ced-3**(**n1286**)* null mutation into the integrated APOE4+APP strain. CED-3 is an executioner caspase that is part of the core apoptotic machinery in worm (Yuan *et al.* 1993). We found that the incidence of both BW and HSN neurodegeneration did not decrease in APOE4+APP strains with or without a mutation in this key apoptotic caspase (Figure 6A-C). Thus, pan-neuronal co-expression of *APOE4* and *APP* appears to cause HSN neurons to degenerate via a mechanism other than apoptosis.

## Discussion

In this study, we present a novel Alzheimer’s disease model in *C. elegans*. Although the worm cannot model all aspects of human AD, our model mirrors three important characteristics: APOE variant-specific modulation of degeneration, age-dependent degeneration, and cell-specific patterned degeneration. In humans, the allele ε4 of *APOE* (*APOE4*) increases the risk as well as the mean age of clinical onset of AD in a copy-dependent manner (Corder *et al.* 1993). The more common *APOE* variant in humans, *APOE3*, does not confer this relative risk. Our results show that *APOE4*, but not *APOE3*, causes degeneration in a subset of neurons in *C. elegans*. *APP* co-expression enhances this *APOE4*-related degeneration of the HSN neurons in *C. elegans*. By contrast, the HSN neurons do not appear to degenerate when *APP* is expressed alone. Similar APOE variant-specific effects have been observed in mouse models. In APOE-target replacement (TR) mouse models engineered on an amyloid-β transgenic background, pathological phenotypes, including amyloid-β deposition, are observed at a higher level in APOE4-TR mice than APOE3-TR mice (Bales *et al.* 2009; Fryer *et al.* 2005). Further, compared to APOE2 and APOE3, APOE4 does not ameliorate neurodegeneration caused by amyloid-β deposition in the glutamatergic tail neurons of another *C. elegans* AD model (Griffin *et al.* 2019). However, studies that show a direct mechanistic link between *APP* and *APOE4* for neurodegeneration are still lacking.

In our *APOE4*-expressing strains, GFP-labeled HSN neurons display neurodegeneration by becoming dim, shrinking, forming blebs, and disappearing in many animals. We also found that the long axonal processes of HSN neurons, which extend anteriorly to synapse onto neurons in the nerve ring, undergo beading. This is reminiscent of how neuronal processes degenerate after laser axotomy (Yanik *et al.* 2006). Indeed, a recently published *C. elegans* AD model describes similar neurodegeneration patterns in the five glutamatergic tail neurons in the presence of Aβ (Griffin *et al.* 2019). In mice, targeted replacement of ApoE with human APOE4 causes dendrites of neurons in the amygdala to shrink compared to the dendrites of APOE3-TR mice (Klein *et al.* 2010). These shared morphological changes in the processes of dying neurons suggest that there might be conserved pathways for degeneration.

Notably, we found that expression of *APOE4* on its own was sufficient to induce significant HSN neurodegeneration and its associated bag-of-worms behavioral phenotype. In contrast, Griffin *et al.* (2019) found that APOE4 expression did not appear to cause neurodegeneration in *C. elegans*. These different results may be explained by our pan-neuronal *vs.* their restricted glutamatergic expression of APOE4. Prevailing hypotheses suggest the interaction between APOE4 and the Aβ fragment drives AD progression. However, a number of studies in mice have shown that APOE4 alone can influence AD progression by affecting numerous, Aβ-independent processes including impaired learning behaviors, reduced synaptic plasticity and neurogenesis, increased neuroinflammation, neurotoxicity of fragmented APOE4, impaired mitochondrial function, and increased tauopathy (Yadong Huang 2011; Yamazaki *et al.* 2016). Many of these processes may be tested in worm. In fact, *C. elegans* contains an ortholog to human tau, PTL-1, that shows 50% sequence conservation in the microtubule-binding repeats (Goedert *et al.* 1996). Future studies may test how *ptl-1* influences APOE4-induced neurodegeneration in our model. One of the prominent characteristics of AD is age-dependent neurodegeneration. Co-expression of *APP* with *APOE4* throughout the nervous system in *C. elegans* enhances the incidence of *APOE4*-related neurodegeneration. Interestingly, the cumulative effect of *APP* and *APOE4* expression manifests more strongly in Day 3 of adulthood, as reproduction declines. This suggests that the additive insult may arise from an age-related process. In patients with Down syndrome who carry an extra copy of *APP*, *APOE4* appears to hasten the onset of AD and accelerate the progression of neurodegeneration (Patel *et al.* 2011; Head *et al.* 2011).

The synergy we observed for enhanced neurodegeneration by combining *APOE4* with *APP* did not extend to an APP-related gene in *C. elegans* called *apl-1*. In fact, we found a modest protective effect of overexpressing an extra copy of *apl-1*. APL-1 is highly homologous to the intracellular portion of APP (Figure 2A), but not the extracellular or Aβ portions of APP (Daigle and Li 1993). Because several clinical trials targeting Aβ have failed to ameliorate cognitive decline, further studies may shift to the role of the APP intracellular domain (AICD). The AICD has been implicated in enhancing AD through its roles in transcriptional activation, pro-apoptotic functions, cytoskeletal modifications, calcium homeostasis modulation, and tau phosphorylation (Müller *et al.* 2008; Chang and Suh 2010; Ghosal *et al.* 2016). In our model, a single extra copy of APL-1 appears mildly protective, but multicopy overexpression of *apl-1* has shown defects in movement, brood size, development, chemotaxis, and learning paradigms (Hornsten *et al.* 2007; Ewald *et al.* 2012). This suggests expression level may also play a role in disease progression. Future studies will need to determine whether neuroprotection against APOE4 stems from the homologous intracellular side and/or other pathways while paying careful attention to expression levels.

In humans, neurodegeneration occurs more extensively in certain regions of the brain, such as the hippocampus and entorhinal cortex (Saxena and Caroni 2011). This aspect is one of the more curious observations in AD patients because APP and APOE4 are both expressed throughout the brain (Huang and Mucke 2012). Our results show that numerous VA and VB cholinergic neurons did not degenerate despite the pan-neuronal expression of *APP* and *APOE4* transgenes and their proximity to the vulnerable VC and HSN neurons. The restricted pattern of *APOE4*-dependent degeneration in worm suggests that there may be cellular and/or molecular characteristics that confer resistance (*e.g.*, VA and VB) or vulnerability (HSNs) to *APOE4*.

Although APOE4 is expressed in both the liver and the nervous system in human (Holtzman *et al.* 2012; Liu *et al.* 2013; Mahley *et al.* 2006), it is unclear whether hepatic, neuronal, or both types of APOE4 contribute to neurodegeneration. We were interested in exploring whether *APOE4*-induced degeneration is tissue-specific in worm. Recent studies have demonstrated clear examples of cell non-autonomous signaling between neuronal and non-neuronal tissue in *C. elegans* (van Oosten-Hawle *et al.* 2013; Taylor and Dillin 2013; Melentijevic *et al.* 2017). For instance, unfolded protein responses, which may underlie neurodegeneration, are interdependently co-activated between the nervous systems and the intestine (van Oosten-Hawle *et al.* 2013; Taylor and Dillin 2013). Additionally, when under stress worm neurons jettison particles extracellularly to become absorbed by coelomocytes (Melentijevic *et al.* 2017). Taken together, these studies suggest that *APOE4* could very well influence neurodegeneration when expressed outside of the neurons. However, after probing expression in both intestine and coelmocytes, our results indicate that *APOE4* mainly induces degeneration when expressed in the nervous system of *C. elegans*. These results are consistent with other work showing the sufficiency of neuronal *APOE4* expression in causing exacerbated cellular or behavioral deficits. For example, in mice neuronal *APP* can cause degeneration and memory deficits when co-expressed with *APOE4* (Bien-Ly *et al.* 2011; Harris *et al.* 2003).

To begin to understand the basis for neurodegeneration in our model, we asked whether degeneration of HSN neurons required apoptosis. CED-3 is the primary executioner caspase that initiates the cascade of apoptosis (Yuan *et al.* 1993). When a *ced-3* null mutation was crossed into the APOE4+APP strain, we saw no reduction of the bag-of-worms phenotype or HSN neurodegeneration. This indicates that apoptosis is likely not involved in *APOE4*-dependent degeneration in *C. elegans*. Other *C. elegans* neurodegenerative models similarly show that *ced-3* and other broad apoptotic mechanisms do not play a role in models of degeneration (*e.g.*, Liachko *et al.* 2010). Necrosis has been previously observed in models of neurodegeneration caused by a number of insults including hyperactive ion channels, increased intracellular Ca^2+^, and protein aggregate-induced stress (Nikoletopoulou and Tavernarakis 2014). Genetic and pharmacological manipulation of necrosis pathways have been previously described in worm, and the drug thapsigargin, a modulator of cytosolic Ca^2+^, has been shown to moderately repress neurodegeneration in worms expressing APOE4 and Aβ (Caraveo *et al.* 2014; Griffin *et al.* 2019). We plan to use these and other methods to elucidate the mechanism of neurodegeneration in our APOE4 model in *C. elegans*. Our model is well-positioned to be used to explore how patterned neurodegeneration may arise in AD and other neurodegenerative diseases.

## References

[bib1] Altun, Z. F., and D. H. Hall, 2018 Reproductive system. In WormAtlas. doi:10.3908/wormatlas.1.17 10.3908/wormatlas.1.17

[bib2] Angelo G., and Van Gilst M. R., 2009 Starvation protects germline stem cells and extends reproductive longevity in *C. elegans.* Science 326: 954–958. 10.1126/science.1178343 19713489

[bib3] Arey R.N., and Murphy C.T., 2017 Conserved regulators of cognitive aging: From worms to humans. Behav Brain Res 322: 299–310. 10.1016/j.bbr.2016.06.035 27329151 PMC5164975

[bib4] Bales K. R., Liu F., Wu S., Lin S., Koger D., 2009 Human APOE isoform-dependent effects on brain beta-amyloid levels in PDAPP transgenic mice. J. Neurosci. 29: 6771–6779. 10.1523/JNEUROSCI.0887-09.2009 19474305 PMC6665579

[bib5] Bien-Ly N., Andrews-Zwilling Y., Xu Q., Bernardo A., Wang C., 2011 C-terminal-truncated apolipoprotein (apo) E4 inefficiently clears amyloid-beta (Abeta) and acts in concert with Abeta to elicit neuronal and behavioral deficits in mice. Proc. Natl. Acad. Sci. USA 108: 4236–4241. 10.1073/pnas.1018381108 21368138 PMC3053957

[bib64] Brenner S. 1974 The genetics of *Caenorhabditis elegans*. Genetics 77: 71–94. 10.1093/genetics/77.1.71 PMC1213120 4366476

[bib6] Cabrejo L., Guyant-Maréchal L., Laquerrière A., Vercelletto M., de la Fournière F., 2006 Phenotype associated with APP duplication in five families. Brain 129: 2966–2976. 10.1093/brain/awl237 16959815

[bib7] Caraveo G., Auluck P. K., Whitesell L., Chung C. Y., Baru V., 2014 Calcineurin determines toxic versus beneficial responses to α-synuclein. Proc. Natl. Acad. Sci. USA 111: E3544–E3552. 10.1073/pnas.1413201111 25122673 PMC4151770

[bib8] Chang K. A., and Suh Y. H., 2010 Possible roles of amyloid intracelular domain of amyloid precursor protein. BMB Rep. 43: 656–663. 10.5483/BMBRep.2010.43.10.656 21034527

[bib9] Chêne G., Beiser A., Au R., Preis S. R., Wolf P. A., 2015 Gender and incidence of dementia in the Framingham Heart Study from mid-adult life. Alzheimers Dement. 11: 310–320. 10.1016/j.jalz.2013.10.005 24418058 PMC4092061

[bib10] Conejero-Goldberg C., Gomar J. J., Bobes-Bascaran T., Hyde T. M., Kleinman J. E., 2014 APOE2 enhances neuroprotection against Alzheimer’s disease through multiple molecular mechanisms. Mol. Psychiatry 19: 1243–1250. 10.1038/mp.2013.194 24492349

[bib11] Conradt B., and Horvitz H. R., 1998 The *C. elegans* protein EGL-1 is required for programmed cell death and interacts with the Bcl-2-like protein CED-9. Cell 93: 519–529. 10.1016/S0092-8674(00)81182-4 9604928

[bib12] Corder E. H., Saunders A. M., Strittmatter W. J., Schmechel D. E., Gaskell P. C., 1993 Gene dose of apolipoprotein E type 4 allele and the risk of Alzheimer’s disease in late onset families. Science 261: 921–923. 10.1126/science.8346443 8346443

[bib13] Corder E. H., Saunders A. M., Risch N. J., Strittmatter W. J., Schmechel D. E., 1994 Protective effect of apolipoprotein E type 2 allele for late onset Alzheimer disease. Nat. Genet. 7: 180–184. 10.1038/ng0694-180 7920638

[bib14] Daigle I., and Li C., 1993 *apl-1*, a *Caenorhabditis elegans* gene encoding a protein related to the human β-amyloid protein precursor. Proc. Natl. Acad. Sci. USA 90: 12045–12049. 10.1073/pnas.90.24.12045 8265668 PMC48122

[bib15] Di Battista A. M., Heinsinger N. M., and Rebeck G. W., 2016 Alzheimer’s Disease Genetic Risk Factor APOE-ε4 Also Affects Normal Brain Function. Curr. Alzheimer Res. 13: 1200–1207. 10.2174/1567205013666160401115127 27033053 PMC5839141

[bib16] Ewald C. Y., Cheng R., Tolen L., Shah V., Gillani A., 2012 Pan-neuronal expression of APL-1, an APP-related protein, disrupts olfactory, gustatory, and touch plasticity in *Caenorhabditis elegans*. J. Neurosci. 32: 10156–10169. 10.1523/JNEUROSCI.0495-12.2012 22836251 PMC3698849

[bib17] Farrer L. A., Cupples L. A., Haines J. L., Hyman B., Kukull W. A., 1997 Effects of age, sex, and ethnicity on the association between apolipoprotein E genotype and Alzheimer disease. A meta-analysis. APOE and Alzheimer Disease Meta Analysis Consortium. JAMA 278: 1349–1356. 10.1001/jama.1997.03550160069041 9343467

[bib18] Frøkjaer-Jensen C., Davis M. W., Hopkins C. E., Newman B. J., Thummel J. M., 2008 Single-copy insertion of transgenes in Caenorhabditis elegans. Nat. Genet. 40: 1375–1383. 10.1038/ng.248 18953339 PMC2749959

[bib19] Fryer J. D., Simmons K., Parsadanian M., Bales K. R., Paul S. M., 2005 Human apolipoprotein E4 alters the amyloid-beta 40:42 ratio and promotes the formation of cerebral amyloid angiopathy in an amyloid precursor protein transgenic model. J. Neurosci. 25: 2803–2810. 10.1523/JNEUROSCI.5170-04.2005 15772340 PMC6725147

[bib20] Ghosal K., Fan Q., Dawson H. N., and Pimplikar S. W., 2016 Tau protein mediates APP Intracellular Domain (AICD)-induced Alzheimer’s-like pathological features in mice. PLoS One 11: e0159435 10.1371/journal.pone.0159435 27459671 PMC4961442

[bib21] Goate A., Chartier-Harlin M. C., Mullan M., Brown J., Crawford F., 1991 Segregation of a missense mutation in the amyloid precursor protein gene with familial Alzheimer’s disease. Nature 349: 704–706. 10.1038/349704a0 1671712

[bib22] Goedert M., Baur C. P., Ahringer J., Jakes R., Hasegawa M., 1996 PTL-1, a microtubule-associated protein with tau-like repeats from the nematode *Caenorhabditis elegans*. J. Cell Sci. 109: 2661–2672. 8937984 10.1242/jcs.109.11.2661

[bib23] Griffin E. F., Caldwell K. A., and Caldwell G. A., 2017 Genetic and Pharmacological Discovery for Alzheimer’s Disease Using *Caenorhabditis elegans*. ACS Chem. Neurosci. 8: 2596–2606. 10.1021/acschemneuro.7b00361 29022701

[bib24] Griffin E. F., Scopel S. E., Stephen C. A., Holzhauer A. C., Vaji M. A., 2019 ApoE-associated modulation of neuroprotection from Aβ-mediated neurodegeneration in transgenic *Caenorhabditis elegans*. Dis. Model. Mech. 12: dmm037218 10.1242/dmm.037218 30683808 PMC6398492

[bib25] Harris F. M., Brecht W. J., Xu Q., Tesseur I., Kekonius L., 2003 Carboxyl-terminal-truncated apolipoprotein E4 causes Alzheimer’s disease-like neurodegeneration and behavioral deficits in transgenic mice. Proc. Natl. Acad. Sci. USA 100: 10966–10971. 10.1073/pnas.1434398100 12939405 PMC196910

[bib26] Head E., Doran E., Mihaela N., Hill M. A., Schmitt F. A., 2011 Plasma amyloid-β as a function of age, level of intellectual disability, and the presence of dementia in Down Syndrome. J. Alzheimers Dis. 23: 399–409. 10.3233/JAD-2010-101335 21116050 PMC3219221

[bib27] Holtzman D. M., Herz J., Bu G., 2012 Apolipoprotein E and apolipoprotein E receptors: normal biology and roles in Alzheimer disease. Cold Spring Harb perspect med. 2: a006312 10.1101/cshperspect.a006312 22393530 PMC3282491

[bib28] Hornsten A., Lieberthal J., Fadia S., Malins R., Ha L., 2007 APL-1, a Caenorhabditis elegans protein related to the human β-amyloid precursor protein, is essential for viability. Proc. Natl. Acad. Sci. USA 104: 1971–1976. 10.1073/pnas.0603997104 17267616 PMC1794273

[bib29] Hsieh J., and Fire A., 2000 Recognition and silencing of repeated DNA. Annu. Rev. Genet. 34: 187–204. 10.1146/annurev.genet.34.1.187 11092826

[bib30] Huang Y., 2011 Roles of apolipoprotein E 4 (APOE4) in the pathogenesis of Alzheimer’s disease: lessons from ApoE mouse models. Biochem. Soc. Trans. 39: 924–932. 10.1042/BST0390924 21787325

[bib31] Huang Y., and Mucke L., 2012 Alzheimer mechanisms and therapeutic strategies. Cell 148: 1204–1222. 10.1016/j.cell.2012.02.040 22424230 PMC3319071

[bib32] Jankowsky J. L., and Zheng H., 2017 Practical considerations for choosing a mouse model of Alzheimer’s disease. Mol. Neurodegener. 12: 89 10.1186/s13024-017-0231-7 29273078 PMC5741956

[bib33] Jonsson T., Atwal J. K., Steinberg S., Snaedal J., Jonsson P. V., 2012 A mutation in APP protects against Alzheimer’s disease and age-related cognitive decline. Nature 488: 96–99. 10.1038/nature11283 22801501

[bib34] Kim J., Jiang H., Park S., Eltorai A. E., Stewart F. R., 2011 Haploinsufficiency of human APOE reduces amyloid deposition in a mouse model of amyloid-β amyloidosis. J. Neurosci. 31: 18007–18012. 10.1523/JNEUROSCI.3773-11.2011 22159114 PMC3257514

[bib35] Klein R. C., Mace B. E., Moore S. D., and Sullivan P. M., 2010 Progressive loss of synaptic integrity in human apolipoprotein E4 targeted replacement mice and attenuation by apolipoprotein E2. Neuroscience 171: 1265–1272. 10.1016/j.neuroscience.2010.10.027 20951774 PMC2991419

[bib36] LaFerla F. M., and Green K. N., 2012 Animal Models of Alzheimer Disease. Cold Spring Harb. Perspect. Med. 2: a006320 10.1101/cshperspect.a006320 23002015 PMC3543097

[bib37] Leung-Hagesteijn C., Spence A. M., Stern B. D., Zhou Y., Su M. W., 1992 UNC-5, a transmembrane protein with immunoglobulin and thrombospondin type 1 domains, guides cell and pioneer axon migrations in *C. elegans*. Cell 71: 289–299. 10.1016/0092-8674(92)90357-I 1384987

[bib38] Liachko N. F., Guthrie C. R., and Kraemer B. C., 2010 Phosphorylation promotes neurotoxicity in a *Caenorhabditis elegans* model of TDP-43 proteinopathy. J. Neurosci. 30: 16208–16219. 10.1523/JNEUROSCI.2911-10.2010 21123567 PMC3075589

[bib39] Liu C. C., Liu C. C., Kanekiyo T., Xu H., and Bu B., 2013 Apolipoprotein E and Alzheimer disease: risk, mechanisms and therapy. Nat. Rev. Neurol. 9: 106–118. 10.1038/nrneurol.2012.263 23296339 PMC3726719

[bib40] Liu C. C., Zhao N., Fu Y., Wang N., Linares C., 2017 ApoE4 accelerates early seeding of amyloid pathology. Neuron 96: 1024–1032.e3. 10.1016/j.neuron.2017.11.013 29216449 PMC5948105

[bib41] Mahley R. W., Weisgraber K. H., and Huang Y., 2006 Apolipoprotein E4: a causative factor and therapeutic target in neuropathology, including Alzheimer’s disease. Proc. Natl. Acad. Sci. USA 103: 5644–5651. 10.1073/pnas.0600549103 16567625 PMC1414631

[bib42] Melentijevic I., Toth M. L., Arnold M. L., Guasp R. J., Harinath G., 2017 *C. elegans* neurons jettison protein aggregates and mitochondria under neurotoxic stress. Nature 542: 367–371. 10.1038/nature21362 28178240 PMC5336134

[bib43] Mello C. C., Kramer J. M., Stinchcomb D., and Ambros V., 1991 Efficient gene transfer in *C. elegans*: extrachromosomal maintenance and integration of transforming sequences. EMBO J. 10: 3959–3970. 10.1002/j.1460-2075.1991.tb04966.x 1935914 PMC453137

[bib44] Mondal S., Hegarty E., Sahn J. J., Scott L. L., Gökçe S. K., 2018 High-Content Microfluidic Screening Platform Used To Identify σ2R/Tmem97 Binding Ligands that Reduce Age-Dependent Neurodegeneration in *C. elegans* SC_APP Model. ACS Chem. Neurosci. 9: 1014–1026. 10.1021/acschemneuro.7b00428 29426225 PMC5955835

[bib45] Müller T., Meyer H. E., Egensperger R., and Marcus K., 2008 The amyloid precursor protein intracellular domain (AICD) as modulator of gene expression, apoptosis, and cytoskeletal dynamics – relevance for Alzheimer’s disease. Prog. Neurobiol. 85: 393–406. 10.1016/j.pneurobio.2008.05.002 18603345

[bib46] Nikoletopoulou V., and Tavernarakis N., 2014 Necrotic cell death in *Caenorhabditis elegans*. Methods Enzymol. 545: 127–155. 10.1016/B978-0-12-801430-1.00006-8 25065889

[bib47] Reiman E. M., Arboleda-Velasquez J. F., Quiroz Y. T., Huentelman M. J., Beach T. G., 2020 Exceptionally low likelihood of Alzheimer’s dementia in APOE2 homozygotes from a 5,000-person neuropathological study. Nat. Commun. 11: 667 10.1038/s41467-019-14279-8 32015339 PMC6997393

[bib48] Patel A., Rees S. D., Kelly M. A., Bain S. C., Barnett A. H., 2011 Association of variants within APOE, SORL1, RUNX1, BACE1 and ALDH18A1 with dementia in Alzheimer’s disease in subjects with Down syndrome. Neurosci. Lett. 487: 144–148. 10.1016/j.neulet.2010.10.010 20946940

[bib65] Prasher V. P., Farrer M. J., Kessling A. M., Fisher E. M., West R. J., 1998 Molecular mapping of Alzheimer-type dementia in Down's syndrome. Ann Neurol. 1998 43: 380–383 10.1002/ana.410430316 9506555

[bib49] Saunders A. M., Strittmatter W. J., Schmechel D., George-Hyslop P. H., Pericak-Vance M. A., 1993 Association of apolipoprotein E allele epsilon 4 with late-onset familial and sporadic Alzheimer’s disease. Neurology 43: 1467–1472. 10.1212/WNL.43.8.1467 8350998

[bib50] Saxena S., and Caroni P., 2011 Selective neuronal vulnerability in neurodegenerative diseases: from stressor thresholds to degeneration. Neuron 71: 35–48. 10.1016/j.neuron.2011.06.031 21745636

[bib51] Schafer, W. R., 2005 Egg-laying, *WormBook*, edited by The *C. elegans* Research Community, WormBook, Available at doi/10.1895/wormbook.1.38.1, http://www.wormbook.org. 10.1895/wormbook.1.38.1 PMC4781431 18050396

[bib52] Stefanakis N., Carrera I., and Hobert O., 2015 Regulatory Logic of Pan-Neuronal Gene Expression in *C. elegans*. Neuron 87: 733–750. 10.1016/j.neuron.2015.07.031 26291158 PMC4545498

[bib53] Taylor R. C., and Dillin A., 2013 XBP-1 is a cell-nonautonomous regulator of stress resistance and longevity. Cell 153: 1435–1447. 10.1016/j.cell.2013.05.042 23791175 PMC4771415

[bib54] Trent C., Tsuing N., and Horvitz H. R., 1983 Egg-laying defective mutants of the nematode *Caenorhabditis elegans*. Genetics 104: 619–647. 11813735 10.1093/genetics/104.4.619 PMC1202130

[bib55] van Oosten-Hawle P., Porter R. S., and Morimoto R. I., 2013 Regulation of organismal proteostasis by transcellular chaperone signaling. Cell 153: 1366–1378. 10.1016/j.cell.2013.05.015 23746847 PMC3955170

[bib56] Ward J. D., 2015 Rapid and precise engineering of the *Caenorhabditis elegans* genome with lethal mutation co-conversion and inactivation of NHEJ repair. Genetics 199: 363–377. 10.1534/genetics.114.172361 25491644 PMC4317648

[bib57] Wu L., and Zhao L., 2016 ApoE2 and Alzheimer’s disease: time to take a closer look. Neural Regen. Res. 11: 412–413. 10.4103/1673-5374.179044 27127474 PMC4829000

[bib58] Yamazaki Y., Painter M. M., Bu G., and Kanekiyo T., 2016 Apolipoprotein E as a therapeutic target in Alzheimer’s disease: a review of basic research and clinical evidence. CNS Drugs 30: 773–789. 10.1007/s40263-016-0361-4 27328687 PMC5526196

[bib59] Yanik M. F., Cinar H., Cinar H. N., Gibby A., Chisholm A. D., 2006 Nerve renegeration in *Caenorhabditis elegans* after femtosecond laser axotomy. IEEE J. Sel. Top. Quantum Electron. 12: 1283–1291. 10.1109/JSTQE.2006.879579

[bib60] Ye S., Huang Y., Müllendorff K., Dong L., Giedt G., 2005 Apolipoprotein (apo) E4 enhances amyloid beta peptide production in cultured neuronal cells: apoE structure as a potential therapeutic target. Proc. Natl. Acad. Sci. USA 102: 18700–18705. 10.1073/pnas.0508693102 16344478 PMC1311738

[bib61] Yi B. J. J., Sahn J. J., Ardestani P. M., Evans A. K., Scott L. L., 2017 Small molecule modulator of sigma 2 receptor is neuroprotective and reduces cognitive deficits and neuroinflammation in experimental models of Alzheimer’s disease. J. Neurochem. 140: 561–575. 10.1111/jnc.13917 27926996 PMC5312682

[bib62] Yoshina S., Suehiro Y., Kage-Nakada E., and Mitani S., 2015 Locus-specific integration of extrachromosomal transgenes in *C. elegans* with the CRISPR/Cas9 system. Biochem. Biophys. Rep. 5: 70–76. 10.1016/j.bbrep.2015.11.017 28955808 PMC5600330

[bib63] Yuan J., Shaham S., Ledoux S., Ellis H. M., and Horvitz H. R., 1993 The *C. elegans* cell death gene *ced-3* encodes a protein similar to mammalian interleukin-1 beta-converting enzyme. Cell 75: 641–652. 10.1016/0092-8674(93)90485-9 8242740

